# Investigation of the Effect of Carbonyl Iron Micro-Particles on the Mechanical and Rheological Properties of Isotropic and Anisotropic MREs: Constitutive Magneto-Mechanical Material Model

**DOI:** 10.3390/polym11101705

**Published:** 2019-10-17

**Authors:** Cintya G. Soria-Hernández, Luis M. Palacios-Pineda, Alex Elías-Zúñiga, Imperio A. Perales-Martínez, Oscar Martínez-Romero

**Affiliations:** 1 Departamento de Ingeniería Mecánica y Materiales Avanzados, Tecnológico de Monterrey, School of Engineering and Science. Av. E. Garza Sada 2501 Sur, Monterrey 64849, NL, Mexico; cintya.soria@tec.mx (C.G.S.-H.); anel.perlaes@tec.mx (I.A.P.-M.); oscar.martinez@tec.mx (O.M.-R.); 2 División de Estudios de Posgrado e Investigación, Tecnológico Nacional de México/Instituto Tecnológico de Pachuca, Carr. México-Pachuca Km 87.5, Pachuca, Hidalgo, Código Postal 42080, Mexico; palacios@itpachuca.edu.mx

**Keywords:** magnetorheological elastomer, stress softening effect, residual strains, stiffness magnetorheological effect, polydimethylsiloxane elastomer, carbonyl iron particles, Helmholtz free energy

## Abstract

This article focuses on evaluating the influence that the addition of carbonyl iron micro-particles (CIPs) and its alignment have on the mechanical and rheological properties for magnetorheological elastomers (MREs) fabricated using polydimethylsiloxane (PDMS) elastomer, and 24 wt % of silicone oil (SO). A solenoid device was designed and built to fabricate the corresponding composite magnetorheological material and to perform uniaxial cyclic tests under uniform magnetic flux density. Furthermore, a constitutive material model that considers both elastic and magnetic effects was introduced to predict stress-softening and permanent set effects experienced by the MRE samples during cyclic loading tests. Moreover, experimental characterizations via Fourier transform infrared (FTIR), X-ray diffraction (XRD), tensile mechanical testing, and rheological tests were performed on the produced MRE samples in order to assess mechanical and rheological material properties such as mechanical strength, material stiffness, Mullins and permanent set effects, damping ratio, stiffness magnetorheological effect (SMR), and relative magnetorheological storage and loss moduli effects. Experimental results and theoretical predictions confirmed that for a CIPs concentration of 70 wt %, the material samples exhibit the highest shear modulus, stress-softening effects, and engineering stress values when the samples are subject to a maximum stretch value of 1.64 and a uniform magnetic flux density of 52.2 mT.

## 1. Introduction

Magnetorheological elastomers (MREs) are materials capable of exhibiting variable stiffness and damping properties, which can be modified by applying an external magnetic field; therefore, these materials are used in several engineering applications such as tunable vibration absorbers, sensing mechanical and magnetic signals [1], to mention a few. Sohoni [2] manufactured anisotropic MREs and found that the alignment of the reinforced particles tends to improve the mechanical properties of the composite material in comparison with those that have an isotropic distribution of the magnetic particles. Later, these findings were confirmed by Li in [3]. In fact, the chain-like structures induced in anisotropic MR materials enhance their dynamic stiffness and damping properties [4]. It is also well-known that when magnetic particles are added into soft or hard elastomeric matrices, the resulting composite MRE exhibit a significant variation of their mechanical properties upon the application of a magnetic induction field i.e., anisotropic MREs with soft matrix showed large magnetorheological effects when compare to those produced with hard elastomeric matrices. For instance, Gong et al. [5] showed that the addition of 20% silicone oil enhances the performance of a magnetorheological material made with 20% of 704 silicone rubber reinforced with 60 wt % of CIPs. Chen and co-workers in [6], investigated the influence that the bare material, external magnetic flux density, temperature, plasticizer, and wt % of carbonyl iron particles have on the properties of the developed MREs. They found that when the MRE is developed by using natural rubber reinforced with 80 wt % of CIPs the shear modulus increases 133% when an external magnetic field of 1 T is applied. Stepanov in [7], noticed that MREs made from a silicone compound called “SIEL” and produced by GNIIChTEOS reinforced with 35% vol. iron microparticles, remember their shape in the presence of magnetic field, i.e., the material deformation is virtually fixed by the magnetic field. They called this effect pseudo-plasticity since the material remembers its shape and there is remarkable increase in the shear loss modulus when the magnetic field is applied.

By using a silicone matrix reinforced with iron particles of 5 and 40 μm, Bose [8] evaluated the influence that que quantity and size of the iron particles have on the mechanical properties of the composite material. They found that for bigger size particles, a larger relative increase in the material storage modulus was attained. The durability properties of MRE materials based on a mixture matrix of cis-polybutadiene rubber (BR) and natural rubber (NR) reinforced with 60 wt % of iron particles were investigated by Zhang et al. in [9]. They performed experimental dynamical tests by using dynamic mechanical analyzer (DMA) tests to study how the storage and loss modulus of the MRE samples vary when subjected to 140,000 loading cycles. Besides, experimental results showed that the storage and loss moduli of all MRE samples tend to decrease with the number of cycles; however, both tend to increase with the aging time. They also found that the magnetorheological (MR) effect increased with the number of loading cycles but this decreased with the aging time when the samples were subjected to 70 °C.

Yu and coworkers [10] showed that the low carbon content in the CIPs has an important role in mechanical performance of MREs. They found that the storage modulus and the MR effect was improved by adding to a 704-silicone rubber matrix, 15, 20, and 65 wt % of CIP with low carbon content. They observed the same tendency with the MRE’s shear storage modulus value when a magnetic field was applied.

Li et al. [11] found that when the quantity of CIPs increases in the polydimethylsiloxane (PDMS) matrix, the composite viscoelastic properties remain almost linear, however, when the fraction of CIPs increases from 60 to 90 wt % the linear viscoelastic range slightly decreases, although the MR effect and the material initial stiffness increase. By adding tactile mutator silicone additive and plasticizer to a matrix silicone material, Stoll [12] found that the resulting magneto-active-elastomer (MAE) with 85 wt % of CIPs exhibited strong magneto-induced change in the storage modulus, resulting in a MR effect 30 times higher than previously reported values for both isotropic and aligned-particles samples.

In an attempt to improve the dispersion of CIPs in a rubbery medium, An and coworkers [13] pretreated the iron particles with (3-aminopropyl) triethoxy silane (APTES) to produce a MRE with a material matrix of natural rubber. The MR effect for the developed MRE with 56 wt % coated CIPs was compared with that of a MRE with uncoated CIPs. They found superior magnetorheological properties for the MRE samples with coated CIPs because of the better affinity that the APTES coating has with the natural rubber matrix. To fabricate high performance MRE, Tian and Nakano [4] subjected the mixed silicone rubber matrix reinforced with 70 wt % of CIP to a magnetic field that was rotated 45° so that the CIPs were forced to be oriented in the field direction. They found that when 15 wt % of silicone oil is added into the silicone mixed, the magnetorheological elastomer properties are better than those of a silicone composite with 0 wt % of silicone oil. Furthermore, they found that because of the 45° particle alignment, the storage modulus had up to 40% superior values in the negative direction opposite to the application of the shear force. Recently, Khairi and co-workers [14] investigated the enhancement of particle alignment in the fabrication of MRE using different concentrations of SO plasticizers and 70 wt % of CIPs. They found that the softening effect of adding 15 wt % of SO improves not only the CIPs incorporation and dispersion for isotropic MRE and particle alignment for producing anisotropic MRE, but it also improves the MR effects of the fabricated material samples.

It is evident that a good understanding of the behavior of MREs is critical to improve their performance and applications, not only to mitigate vibrations via the introduction of MRE components in the design of vibration absorbers, isolators, mounts, vehicle seats, and vibration suspension systems, etc. [15], but also to promote their use in other industrial sectors. In this sense, MREs reinforced with hard magnetic particles have been developed for soft continuum robots capable of omnidirectional steering and navigation [16], with a carrying capacity of 100 times its own weight [17], and with enhanced magnetorheological properties that allow them to swim, to climb, to roll and walk, to jump, and to crawl within narrow tunnels [18]. Therefore, a good understanding of their modeling to predict long-term durability and performance is highly desirable to widen the application of these materials.

Thus, this work focused first on experimentally evaluating the mechanical and rheological properties that the addition of 20, 27, 45, 63 and 70 wt % of CIPs and its alignment have for MREs fabricated by considering polydimethylsiloxane (PDMS) elastomer matrix material, and 24 wt % of silicone oil (SO). Secondly, we introduced a mathematical model capable of predicting the material response behavior when subjected to uniaxial cyclic loads, under the influence of magnetic effects. To collect experimental data needed to validate the accuracy of the proposed MRE constitutive equation, a solenoid device was built to generate a uniform magnetic field that allowed us not only to fabricate MREs material samples, but also to perform cyclic uniaxial tests subjected to a uniform magnetic flux density. In this sense, the solenoid characterization was performed using a longitudinal probe attached to a Gaussmeter and fixed to a vertical support that was able to move vertically to measure the magnetic flux density. Also, finite element simulations were conducted to identify the solenoid longitudinal axis range of uniform magnetic flux density to set the maximum displacement for the MRE samples inside the solenoid device during uniaxial cyclic tests.

Experimental results via Fourier transform infrared (FTIR), X-ray diffraction (XRD), tensile and cyclic mechanical testing, and rheological tests confirmed that particle alignment and the wt % of CIPs influence the mechanical and rheological properties of the fabricated MREs.

## 2. Materials and Methods

The materials used to manufacture the elastomeric-composite were dimethyl-silicone oil (SO), which has a viscosity of 0.25 Pa s, and silicone rubber Ecoflex00-10, purchased from Mörph Industries (México City, México). Spherical carbonyl iron particles with an average size of 3.0 µm were purchased from Sigma-Aldrich (México City, México). The composite materials were prepared by following the procedure steps described in [19] and by considering the following concentrations of magnetic iron micro-particles: 20, 27, 45, 63 and 70 wt %. First, the magnetic particles were immersed in 24 wt % of SO and mixed for a couple of minutes, then the SR was added. All the ingredients were mixed at room temperature during 5 min. The homogeneous mixture was placed into a mold and the curing process was carried on at room temperature during 12 h and under vacuum conditions to avoid porosity. During the first 30 min of the curing process, the reinforced samples were exposed to a magnetic flux density of 52 mT.

### 2.1. Solenoid Characterization and Particle Alignment

**Figure 1:**
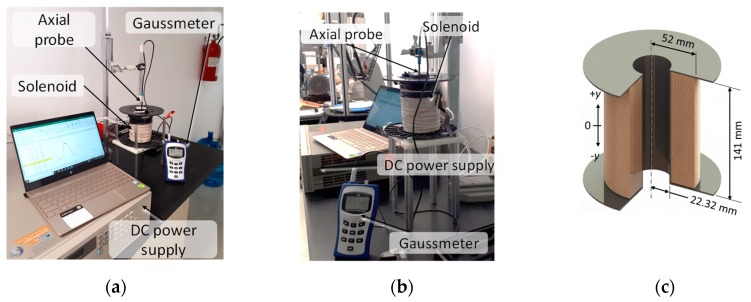
(**a**,**b**) Test rig for solenoid characterization, a Gaussmeter BELL-5170 with longitudinal probe is used to measure the magnetic flux density along the solenoid longitudinal *y* axis. Figure (**c**) depicts solenoid geometrical characteristics, this electromagnet has 3600 wire turns and an electrical resistance of 15 Ohms.

A solenoid was built to generate the magnetic field to be used during the fabrication and experimental sample tests. The solenoid characterization was performed using a longitudinal probe attached to a Gaussmeter and fixed to a vertical support that was able to move vertically to measure the magnetic flux at different longitudinal positions, Figure 1a,b show the experimental set-up, and Figure 1c shows the solenoid dimensions.

The solenoid and its surrounding air were modeled in a finite element computer package ANSYS (Canonsburg, PA, USA) to estimate magnetic flux density inside and around the built solenoid device. A 2D-Axisymmetric model using a 4-node element with one degree of freedom at each node was used. The computational domain has 1120 elements and 296 nodes. The boundary condition considered was the flux parallel condition, which defines the magnetic field lines parallel to the boundary surface for all the external surfaces of the computational domain. A current density of $J=\left\{0.38,\;0.77,\;1.17,\;1.61\right\}{\times 10}^{6}\;A/m^{2}$, which corresponds to the 3600 solenoid turns, with applied current of I={0.53, 1.06, 1.61, 2.21} A were used as the solenoid boundary conditions. Relative permeability $\mu_{r}$, for the surrounding air and for the solenoid copper was considered as 1.

**Figure 2:**
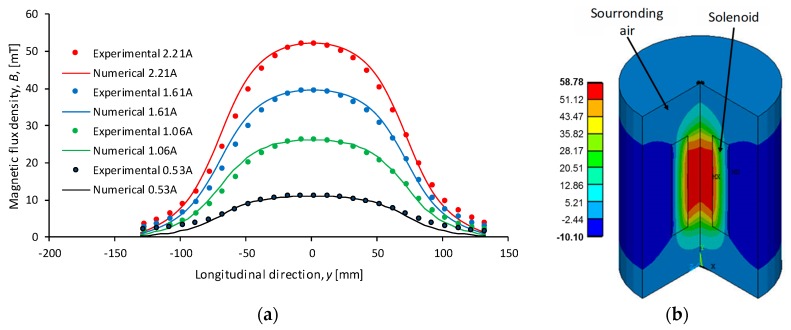
(**a**) Comparison between experimental measurements and numerical results of the magnetic flux density along the solenoid longitudinal axis *y*. As expected, a linear relationship between the electrical current applied to the solenoid and flux density is observed. (**b**) Computed magnetic flux density (mT) in the solenoid and surrounded air, these results were obtained numerically by solving the Maxwell equations, using ANSYS finite element method software.

The simulation results are shown in Figure 2. The value of the magnetic flux density along the solenoid *y* longitudinal axis is shown in Figure 2a, while Figure 2b depicts the ANSYS numerical estimated magnetic flux density inside and around the built solenoid device. Notice from Figure 2 that this electromagnet is capable of delivering a uniform magnetic flux density with only a 4% variation along its longitudinal axis in the range of −25≤y≤25 mm. In this region, it is key to have adequate control on both the material fabrication and during the tensile test of the MRE samples.

**Figure 3:**
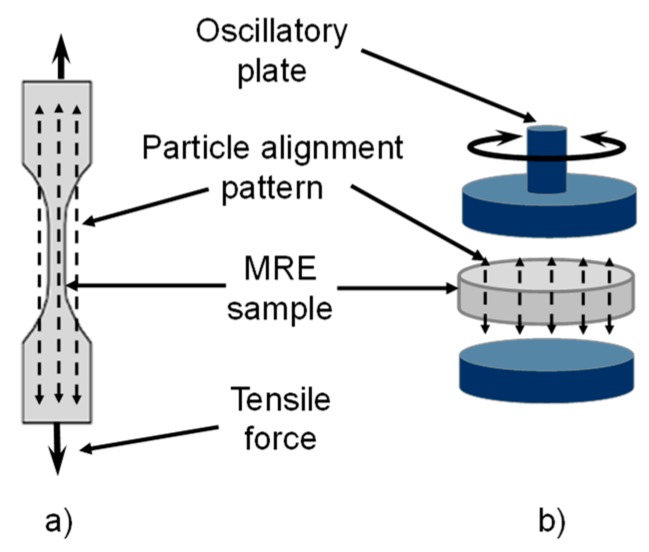
During the polydimethylsiloxane (PDMS) curing and manufacture process, the samples were exposed to a magnetic flux density of 52 mT to produce a specific magnetic micro particle arrangement. (**a**) Particle alignment along the longitudinal axis of the manufactured tensile samples, and (**b**) particle arrangement inside the rheological test samples.

The particle alignment inside the MREs was carefully controlled during the manufacturing process. Figure 3 illustrates the desired particle distribution inside the tensile and rheological samples.

### 2.2. Fourier Transform Infrared

Fourier transform infrared (FTIR) equipment from Perkin-Elmer Frontier with an attenuated total reflectance (UATR) accessory was used to carry out the infrared (IR) analysis. The IR spectra of the elastomer samples were measured in the interval range of 4000 to 400 cm^−1^ with a resolution of 8 cm^−1^, and by considering an average of 16 scans.

### 2.3. X-Ray Diffraction (XRD)

The XRD measurements of elastomer samples were carried out using a PanAnalytical X’Pert Pro PW1800 diffractometer with a scanning rate of 2°/min and by using Cu Kα radiation. The system was operated at 45 mA, 40 kV, and the XRD data were collected in the 2θ range from 10° to 85°.

### 2.4. Morphology and Particle Distribution

The morphology and particle distribution were observed by scanning electron microscopy (SEM, Evo MA 25, Carl Zeiss, Oberkochen, Germany). For the morphology, a small amount of CIP powder was deposited on carbon tape and observed using an accelerating voltage of 20.00 kV and a working distance of 9.0 mm. To obtain the particle size distribution, around 200 particles recorded by SEM were used to estimate the particle mean size distribution by Digimizer 4.6.1 software (MedCale Software Ltd, Ostend, Belgium). With respect to the particle distribution in the elastomer, a cross section of the samples was made and the surface was washed with 50% isopropanol. Subsequently, the samples were observed by SEM using an accelerating voltage of 10 KV and a working distance of 12 mm.

### 2.5. Tensile Tests

**Figure 4:**
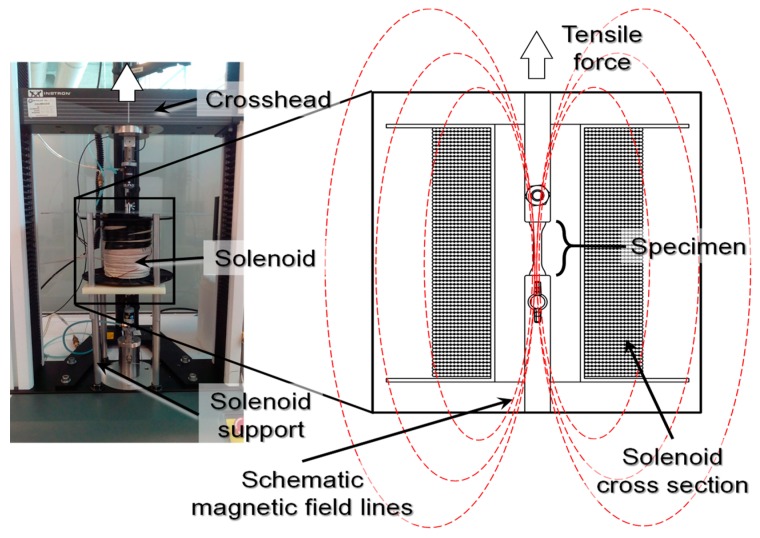
Tensile test set up and its schematic magnetic field applied on the magnetorheological elastomer (MRE) samples, according to the solenoid design and desire experimental measurements. The maximum magnetic field variation along the specimen is only 4% due to the solenoid length relative to the tensile sample material length. Additionally, the experimental set up allows the magnetic field lines to be parallel to the micro particle arrangement inside the material sample.

The tensile strength of the MREs was measured in an INSTRON 3365 universal testing machine at room temperature. The specimens have a dumbbell-shaped geometry according to the specification norm ISO37-2011. The MRE stiffness was measured during tensile testing of material samples by applying an extension of 20 mm with a crosshead rate of 200 mm·min^−1^. As shown in Figure 4, the test set up configuration is prepared in such a way to ensure that the applied magnetic field was parallel to the particle alignment of the MRE (see Figure 4). At least 50 mm of the sample length was kept inside the solenoid during the application of a uniform magnetic flux density. It is important to mention that a Fe–Si steel sheet metal, used as a magnetic shield, was installed to protect the tensile machine load cell and to avoid spurious data. The magnetic flux density was varied from 0–52 mT.

### 2.6. Rheological Test

**Figure 5:**
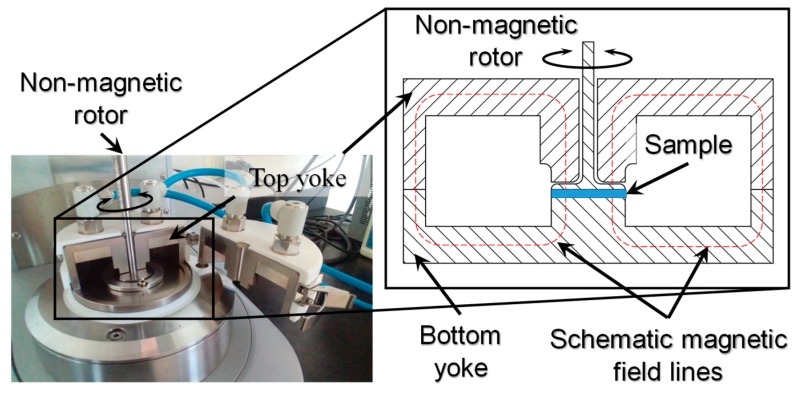
Electromagnetic apparatus installed on the Anton Paar MCR301 Rheometer. This device produces magnetic field lines parallel to the axis of the oscillatory plate. Magnetic field lines are represented schematically and their direction with respect to the sample material are depicted.

To measure the MRE samples rheological properties under the influence of a magnetic field with a parallel-plate rotor configuration, an advanced commercial rheometer (Model: MCR301, Anton Paar, Austria) was used. The measurements of the storage modulus *G*’ and the loss modulus *G”* were performed on cylindrical samples of 10 mm of diameter and 1 mm gap in the regime of dynamical oscillations under controlled strain and frequency. As illustrated in Figure 5, a composite material sample was placed between the base and the rotating disk. The parallel-plate rotor was set to have oscillatory shear mode motion with a frequency of 1 Hz and tuned to have a maximum shear strain of 0.1%. A magnetic field was induced by an electromagnet with magnetic field lines perpendicular to the surface of the MRE sample and parallel to the CIP′s. See Figure 3 and Figure 5. By adjusting the DC power supply from 0 to 5 A, the range of the applied magnetics flux density to the MREs sample was in the interval values of 0–1 T. All measurements were performed at a constant temperature of 20 °C.

## 3. A Stress-Softening Magnetorheological Constitutive Material Model

The available constitutive material models for magnetoactive materials do not considered stress-softening (Mullins) effects. Therefore, we developed a material model that takes into account finite deformations and magnetic field effects. Since most MREs are anisotropic in nature here, we used the “isotropized” Helmholtz free energy density for composite hyperelastic materials developed in [20], and added the magnetic effects to it to have an energy density expression that could be used to develop a stress-stretch constitutive material model that accounts for the mechanical effects induced by applied magnetic fields.

Let us first begin by recalling the “isotropized” Helmholtz free energy density expression for composite hyperelastic materials [20,21]:

(1) $$W^{elastic}=(1-f)W_{iso}(I_{1})+f\left(\frac{A_{1}}{3}(I_{1}-3)+\frac{A_{2}}{9}{\left(I_{1}-3\right)}^{2}-\frac{2A_{1}}{3}\ln\sqrt{I_{3}}\right)$$

where *A*_1_ and *A_2_* are materials constants, *f* is the equivalent anisotropic volumetric fraction, *W*_iso_ (*I*_1_) is the strain energy density that characterizes the isotropic virgin material contribution, and *I*_1_ and *I*_3_ are deformation invariants given as:

(2) $$I_{1}=\mathrm{tr}C,\;I_{3}=\det C,\;C=F^{T}F,\;\mathrm{and}\;F(X)=\partial \kappa (Χ)/\partial X.$$

Here, **F** and **C** are the gradient and the left Cauchy-Green deformation tensors, respectively, **x =** **κ**(***X***) denotes the position vector describing the location of point *P* in the current configuration, while ***X*** denotes the position vector of point *P* in the reference configuration.

To add the work done by magnetic effects to the elastic one, the first and second thermodynamic laws, as well as their corresponding potential relationships for magnetic media are considered. Based on these laws and potentials, it is concluded that for an applied magnetic flux density along a principal deformation axis, the magnetic energy density is given by [22,23,24,25,26,27,28]:

(3) $$W^{magnetic}(F,B)=-\frac{1}{2\mu }FB\cdot B,$$

where **B** is the magnetic flux density vector, *µ* is the material permeability constant, and **F** is the usual deformation gradient tensor.

Adding expressions (2) and (3) provides the total Helmholtz free energy density for the magnetic elastomeric material:

(4) $$W_{T}=(1-f)W_{iso}(I_{1})+f\left(\frac{A_{1}}{3}(I_{1}-3)+\frac{A_{2}}{9}{\left(I_{1}-3\right)}^{2}-\frac{2A_{1}}{3}\ln\sqrt{I_{3}}\right)-\frac{1}{2\mu }FB\cdot B$$

Boyce and Arruda in [29], Steinmann et al. in [30] and Elías-Zúñiga and Beatty in [31] provide several micromechanical and phenomenological models to describe the form that the strain energy density term *W*_iso_(*I*_1_) can assume. However, here we adopt the micromechanical model that comes from statistical mechanics provided by Elías-Zúñiga and Beatty in [31], since this non-Gaussian expression has BEEN shown to provide good description of experimental data. Therefore, term *W*_iso_(*I*_1_) of Equation (4) is assumed to be given by the amended-non-Gaussian strain energy density expression:

(5) $$W_{iso}(I_{1})=G\left[N_{8}\left(\beta \lambda_{r}+\ln\left(\frac{\beta }{\sinh\beta }\right)\right)-\ln\left(\frac{\beta }{\lambda_{r}}\right)\right]+c_{1}$$

where *λ*_r_ represents the relative chain-stretch given by:

(6) $$\lambda_{r}=\frac{\lambda_{chain}}{\sqrt{N_{8}}},\;\lambda_{chain}=\sqrt{\frac{I_{1}}{3},}$$

*N*_8_ is the material chain number of links, $\beta =L^{-1}(\lambda_{r})$ is the inverse of the Langevin function L(β), defined as

(7) $$\lambda_{r}=L(\beta )\equiv \coth\beta -\frac{1}{\beta },$$

*c*_1_ is an energy constant, and *G* is the material shear modulus. Thus, Equation (4) can be written as:

(8) $$\begin{array}{l} W_{T}=(1-f)\left(G\left[N_{8}\left(\beta \lambda_{r}+\ln\left(\frac{\beta }{\sinh\beta }\right)\right)-\ln\left(\frac{\beta }{\lambda_{r}}\right)\right]+c_{1}\right)+\\ f\left(\frac{A_{1}}{3}(I_{1}-3)+\frac{A_{2}}{9}{\left(I_{1}-3\right)}^{2}-\frac{2A_{1}}{3}\ln\sqrt{I_{3}}\right)-\frac{1}{2\mu }FB\cdot B \end{array}$$

Thus, the stress-stretch equation that allow us to compute the corresponding Cauchy stress components *T*_j_ are given by:

(9) $$T_{j}=-p+\lambda_{j}\frac{\partial W}{\partial \lambda_{j}},\;j=1,\;2,\;3\;(\mathrm{no}\;\mathrm{sum}),$$

where *p* is an arbitrary pressure that can be eliminated by subtracting *T*_k_ from *T*_j_:

(10) $$T_{j}-T_{k}=\left\{\left(1-f)ℵ+\frac{2f}{3}\left(A_{1}+\frac{2A_{2}}{3}(I_{1i}-3)\right)-\frac{1}{2\mu }(B_{j}^{2}-B_{k}^{2}\right)\right\}(\lambda_{j}{}^{2}-\lambda_{k}{}^{2})$$

*j* ≠ *k* = 1, 2, 3 (no sum), and

(11) $$ℵ=\frac{G}{3\lambda_{r}}\left[\beta +\frac{1}{N_{8}}\left(\frac{1}{\lambda_{r}}-\frac{1}{\beta \left(1-\lambda_{r}{}^{2}-\frac{2\lambda_{r}}{\beta }\right)}\right)\right].$$

Furthermore, stress softening and permanent set effects that magnetorheological elastomers experience can be predicted by modifying the material model introduced in [32] to add magnetic effects. This provides the following constitutive material model that accounts for mechanical effects induced by applied magnetic fields:

(12) $$\begin{array}{l} \tau_{j}-\tau_{k}=[\left\{\left(1-f)ℵ+\frac{2f}{3}\left(A_{1}+\frac{2A_{2}}{3}(I_{1i}-3)\right)-\frac{1}{2\mu }(B_{j}^{2}-B_{k}^{2}\right)\right\}(\lambda_{j}{}^{2}-\lambda_{k}{}^{2})+\\ \frac{G}{2C}(\lambda_{j}f_{j}(\lambda_{1},\lambda_{2},\lambda_{3})-\lambda_{k}f_{k}(\lambda_{1},\lambda_{2},\lambda_{3})]e^{-b\sqrt{\left(M-m)(m/M\right)}}, \end{array}$$

where *j* ≠ *k* = 1, 2, 3 (no sum). Here,

(13) $$f_{j}(\lambda_{1},\lambda_{2},\lambda_{3})=\frac{\partial \sum_{a=1}^{3}{\left(\lambda_{\max a}^{n}-\lambda_{a}^{n}\right)}^{2}}{\partial \lambda_{j}},$$

*C* is a positive material constant, *b* is a material softening parameter, *n* is a fitting parameter that in general take the value of one, *λ*_a_ are the principal stretches, *λ*_maxa_, *a* = 1, 2, 3 are the maximum principal stretch values at which unloading begins on the virgin loading path, and *m* and *M* for simple uniaxial extension, are given by the following equations:

(14) $$m=\sqrt{\lambda^{4}+2\lambda^{-2}},\;\mathrm{and}\;M=\sqrt{\lambda_{\max}^{4}+2\lambda_{\max}^{-2}}$$

Of course, the engineering stress tensor **σ** can be obtained by using the relationship $\sigma =TF^{-1}$.

The accuracy of the proposed constitutive material models (10) and (12) will be addressed when compared with experimental data in Section 4.7.

## 4. Results

### 4.1. Particle Alignment

**Figure 6:**
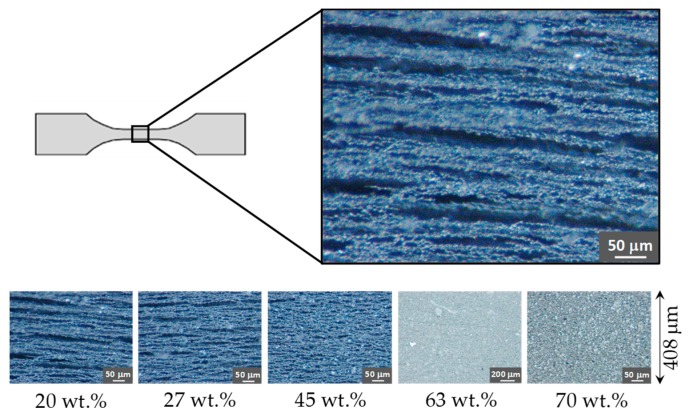
Illustration of carbonyl iron micro-particles (CIPs) inside the tensile test specimens for different particle concentrations, the material samples with 20, 27 and 45 wt % of carbonyl microparticles content have an alignment parallel to the longitudinal sample direction. For material samples with 63 and 70 wt % of CIPs microparticles content, the particles are not aligned. This is because of the viscosity of the uncured polymer, mixed with the magnetic microparticles, increases significantly for material samples with 63 and 70 wt % of CIPs, and the magnetic force field applied during the polymer curing process is not strong enough to produce any particle arrangement.

**Figure 7:**
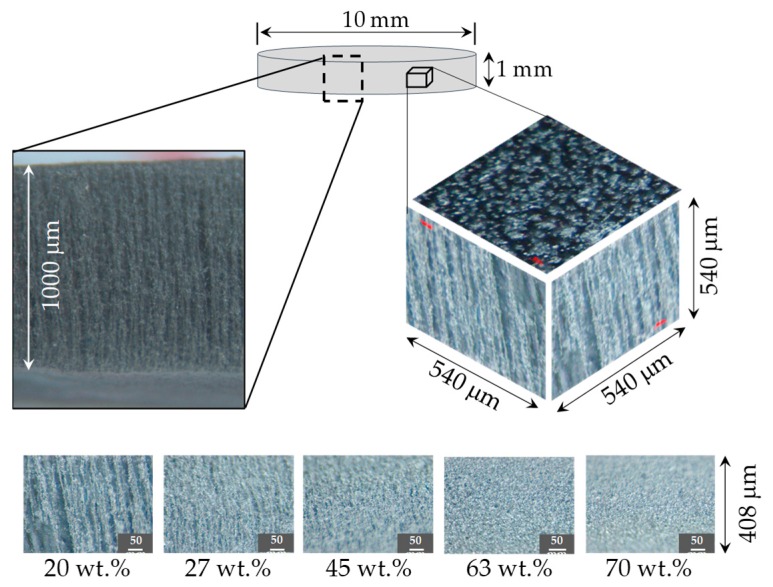
Microphotographs of rheological material samples reinforced with carbonyl iron microparticles. Particle alignment was observed in the material samples for 20, 27 and 45 wt % of CIPs concentrations. However, for specimens with 63 and 70 wt % of CIPs, no particle patterns were observed after exposing the material samples to magnetic flux densities during the composite material manufacturing process.

To verify the particle alignment, the samples were analyzed by optical microscopy. Figure 6 and Figure 7 show the composite materials particle distribution of the material samples used to perform the tensile and rheological characterization test, respectively. These measurements confirmed that the particle alignment is parallel to the longitudinal axis of the material specimens. Notice from Figure 6 and Figure 7 that when the content of CIPs exceeds 63 wt %, particle alignment was not achieved because of the saturation of CIPs. In other words, during their manufacturing process, the magnitude of the induced magnetic forces is not enough to overcome the viscosity forces of the produced composite materials.

### 4.2. Particle Morphology

**Figure 8:**
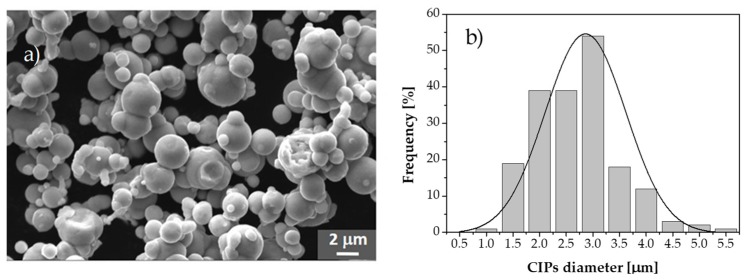
(**a**) Image obtained from SEM equipment. Notice the spherical like-form of the CIPs used to reinforce the PDMS elastomer. (**b**) Based on the Digimizer 4.6.1 software, the particle size distribution obtained from several measurements is in the range of 1–5.5 µm with and average diameter of about 3 µm, which agrees with the supplier technical specifications.

Based on images obtained from SEM measurements, the morphology and size distribution of the CIPs are shown in Figure 8. Notice from Figure 8a that the majority of the CIPs have a spherical shape-form with an average diameter of about 3 µm as shown in Figure 8b.

**Figure 9:**
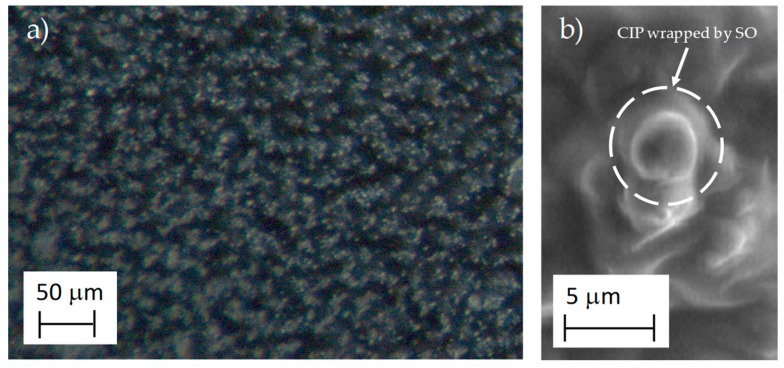
Particle dispersion in the elastomer with 45 wt % of CIPs: (**a**) homogenous CIPs distribution, and (**b**) image that shows a CIP wrapped in the PDMS matrix.

Figure 9a shows the optical microscopy image of the structure of the magnetorheological elastomer. Notice the well dispersed CIPs in the PDMS matrix. The SEM image in Figure 9b confirms that the CIPs are wrapped in PDMS matrix without phase separation, which demonstrates the good compatibility between PDMS and CIPs, which is mainly due to the addition of SO since this increases the plasticity and fluidity and influences the distribution of internal stress in the material [33]. Therefore, it is concluded that the presence of the SO in the magnetorheological elastomer formulation enhance the homogeneous distribution of the CIPs in the silicone rubber matrix.

### 4.3. FTIR Analysis

**Figure 10:**
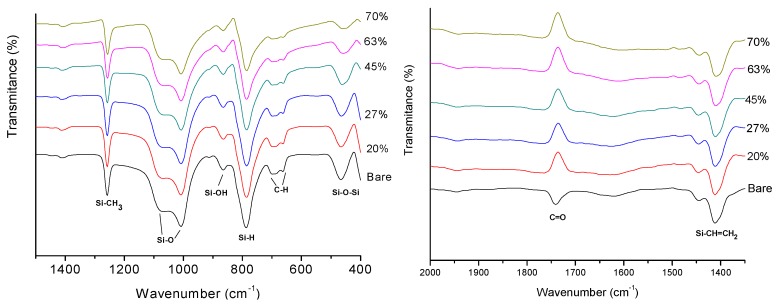
FTIR spectra of anisotropic elastomers of PDMS made with 20%, 27%, 45%, 63% and 70% of CIP′s.

The FTIR spectra of the anisotropic elastomers of PDMS (CH_3_ (Si (CH_3_)_2_O)_n_Si(CH_3_)_3_) made with 20, 27, 45, 63 and 70 wt % of carbonyl iron particles are shown in Figure 10. In 465.13 cm^−1^ a stretching vibration of Si–O–Si was detected, while at the wave numbers of 663.49, 687.66 and 698.05 cm^−1^ the vibrational mode of the C–H was detected [34,35]. It is also observed in Figure 10 that the asymmetric stretching and bending vibration modes that correspond to the Si–H and Si–OH bonds are located at the wave numbers of 787.37 and 911.88 cm^−1^, respectively [34,36,37]. Notice that the signals intensity of the Si–O–Si, C–H, Si–H and Si–OH decrease as the wt % of CIPs increases. This spectral behavior was also observed in [37] at increasing wt % of silica during the synthesis of magnetic nanoparticles. Bands around 1100 cm^−1^ are attributed to the stretching vibration of Si–O, the detected band at 1257.83 cm^−1^ evidence the vibration of Si–CH_3_ and the band 1411.7 cm^−1^ to the Si–CH=CH_2_ vibration. In these three signals there is no significant difference between the spectra of the bare sample and the composite material samples. It was found that at the value of 1766.26 cm^−1^ the bare material presents a signal corresponding to the vibration of the group C=O, while the composite material samples with aligned CIPs maintain the signal in the same wave, but do not absorb. This effect can be attributed to the magnetic field used to align the particles during the composite manufacturing process. The magnetic forces favored the electron donation of the iron particle to the carbonyl antibonding orbitals. This interaction is called metal-binder bond; it neutralizes the charge of the carbonyl and does not interact with the infrared wavelength [38]. The FTIR spectra helps to identify the chemical interaction between the carbonyl groups of the PDMS with the iron particles.

### 4.4. X-Ray Diffraction (XRD)

**Figure 11:**
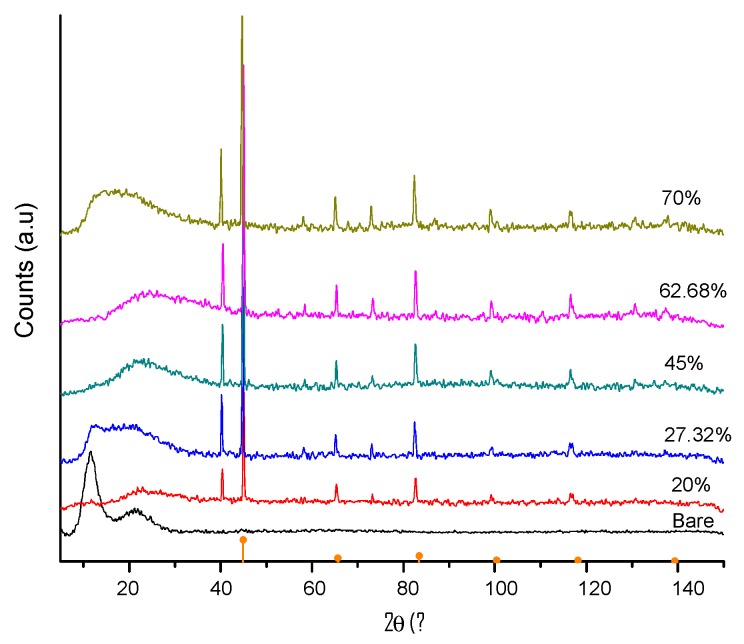
Diffraction pattern (JCPDS 06-0696) of the composite material samples made for the CIPs concentrations of 20, 27, 45, 63 and 70 wt %.

After performing the XRD measurements in the developed material samples, it was found that silicone rubber shows two broad peaks at 2Ɵ values of 12° and 23°, which is consistent with the amorphous nature of silicon rubber (Figure 11). Meanwhile, the iron particles have a well-defined crystalline structure that is corroborated by the intense peaks that appear at 44.7°, 65°, 82.3°, 98.9°, and 116.4° in 2Ɵ of the diffraction pattern of α-Fe (JCPDS 06-0696). These peaks correspond to the *hkl* planes (110), (200), (211), (220) and (319). According to the XRD patterns, the composite elastomer constituents are based on polydimethylsiloxane and CIPs, since its characteristic crystallographic peaks are well defined. This technique allowed us to corroborate the information obtained by FTIR analysis. A diffraction pattern at 40° was found and it does not correspond to the polymer or the particles, it probably corresponds to the C=O–Fe interaction favored by the magnetic field used for the alignment.

### 4.5. Tensile Test Results

**Figure 12:**
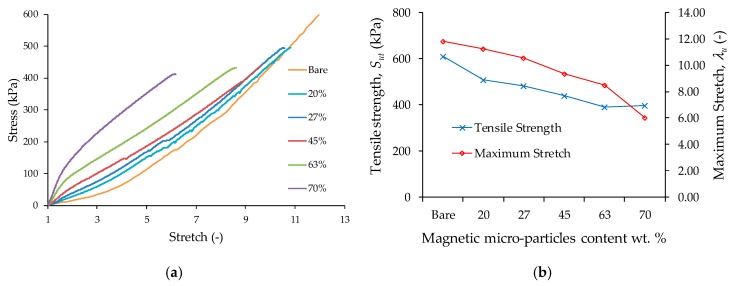
(**a**) Tensile test curves for the bare polymeric matrix sample and for composite material samples with 20, 27, 45, 63 and 70 wt % of CIPs, as the percentage of magnetic microparticles inside the material increases, the material stiffness increases. Also, there is a significant change in the material behavior, the bare and low CIP content (20 and 27 wt %) materials exhibit a nonlinear stiffening behavior, while the materials with high content of magnetic particles (45, 63, and 70 wt %) in the composite material exhibit a nonlinear softening behavior. (**b**) Tensile strength and maximum stretch for the samples shown in (**a**): as the CIP content in the composite material increases, both the tensile strength and maximum stretch decrease.

Tensile tests were performed in PDMS elastomer and for the composite material samples. The results plotted in the curved shown in Figure 12a were based on the average value obtained by performing the experimental test on five specimens of the same material. Notice from Figure 12b that for increasing CIPs concentrations, the material tensile strength decreases when the samples are subjected to large deformations, in fact, a reduction in the strength resistance of about 20% is achieved when comparing the samples made with 20 wt % with respect to those made with 70 wt % of CIPs.

**Figure 13:**
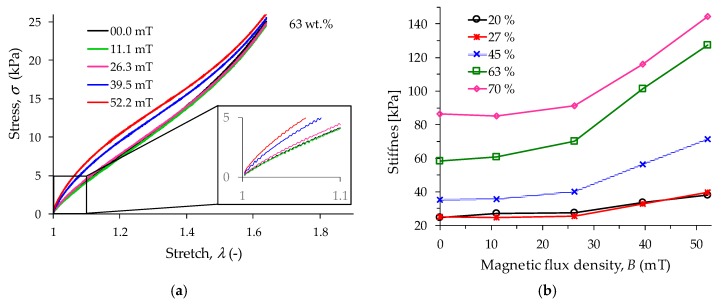
(**a**) Experimental tensile test curves for the composite material with 63 wt % of CIPs were obtained when subjecting the material samples to different magnetic flux intensity values. (**b**) MREs stiffness values obtained from the tensile test with variable magnetic flux density from 0 to 52.2 mT.

**Figure 14:**
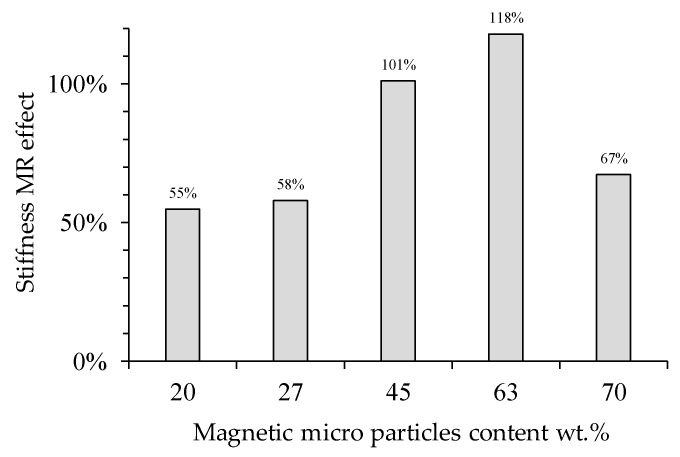
The stiffness magnetorheological (SMR) effect obtained for the composite materials when a 64 mT magnetic flux density is induced under static tensile load conditions. Here, the SMR effect was computed using the material stiffness under the magnetic flux density action with respect to those values obtained when the material samples were tested without a magnetic flux density.

Figure 13 illustrates the influence that the magnetic field has on the composite material stiffness during simple extension tensile tests, in which it is evident that all composite materials exhibit an increment in the stiffness as the magnetic field increases. Without magnetic field, the sample with 20 wt % of CIPs exhibits a stiffness value of 38.1 kPa, while the sample with 70 wt % has a stiffness value of 86.30 kPa which is 127% higher when compared to the previous one. To emphasize the influence that the magnetic flux density has on the material stiffness, in this article, the stiffness magnetorheological (SMR) effect is defined as the ratio of the magneto-induced stiffness modulus and initial material stiffness value. Therefore, the SMR effect is defined as:

(15) $$SMR=\frac{\left(k_{\max}-k_{st}\right)}{k_{st}}100\%$$

where *k*_st_ describes the static stiffness material value without magnetic flux density, *k*_max_ is the maximum stiffness value collected when the material is subjected to a uniform magnetic flux density. Thus, when a 52 mT of magnetic flux density is induced on the material samples, the one with 63 wt % of CIPs shows the highest percentage of SMR effect value improvement, as shown in Figure 14.

### 4.6. Rheology Test Results

The storage modulus recorded in the developed material samples were recorded with and without an induced magnetic field. In fact, during rheological tests, it was found that the recorded storage modulus for the material samples without induced magnetic field was 4.53 kPa for a composite material with 20 wt % of CIPs, and was 24.1 kPa for the sample reinforced with 70 wt %, which is 432% higher than that recorded from the previous material sample with 20 wt % of CIPs.

**Figure 15:**
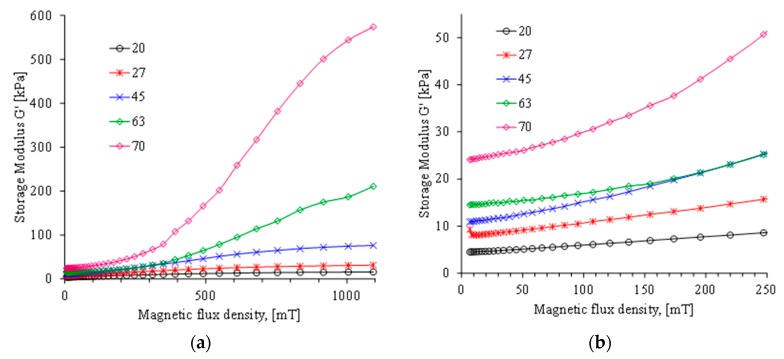
(**a**) Storage modulus behavior for the MRE material samples with 20, 27, 45, 63, and 70 wt % under the action of a variable magnetic flux density from 0 to 1093 mT. (**b**) Detailed view of the material behavior for magnetic flux density range from 0 to 250 mT.

**Figure 16:**
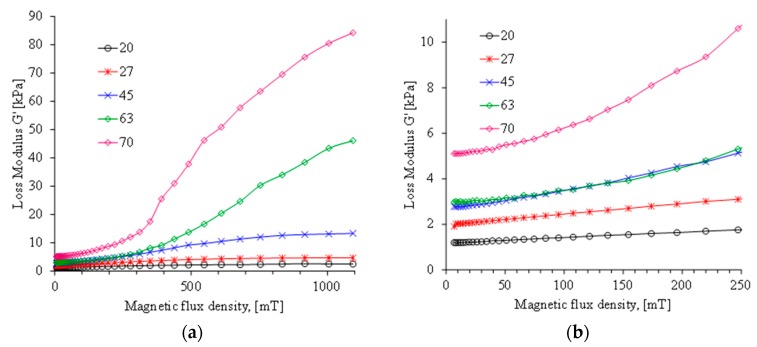
(**a**) Loss modulus behavior for the MRE material samples with 20, 27, 45, 63, and 70 wt % under the action of a variable magnetic flux density from 0 to 1093 mT. (**b**) Detailed view of the material behavior for magnetic flux density range from 0 to 250 mT.

When a uniform magnetic field is induced, the influence of the magnetic field on the MREs dynamic modulus was also measured. Figure 15 and Figure 16 illustrate the variation of the storage and loss modulus when a uniform magnetic field is induced on different MREs samples. Notice that the material sample with 70 wt % of CIPs is the one that exhibits the highest storage and loss modulus values.

**Figure 17:**
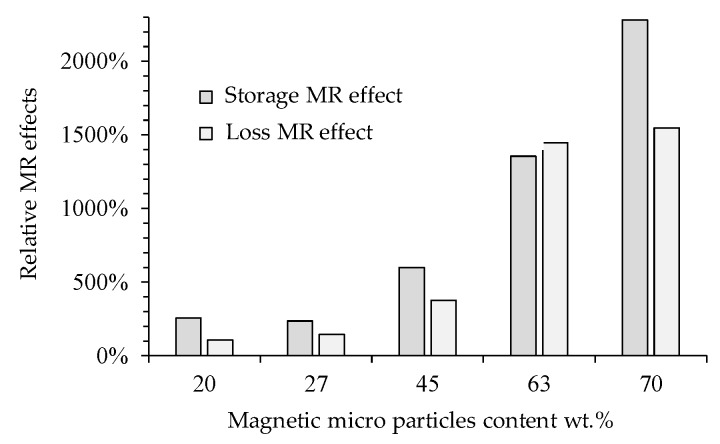
The maximum relative MR effects achieved in the storage and loss moduli for different MRE samples. The MR effects for the storage and loss moduli were computed when a magnetic flux density of 1093 mT was applied with respect to those values obtained when the material samples were subjected to rheology tests without the application of a magnetic flux density.

As expected, when a magnetic field is induced on the MREs samples, the relative storage and loss MR effects [10] increase with respect to those values recorded in the MREs samples without magnetic field, as shown in Figure 17. Notice a significant change in the relative MR effects of the complex modulus in the sample with 70 wt % of magnetic microparticles.

**Figure 18:**
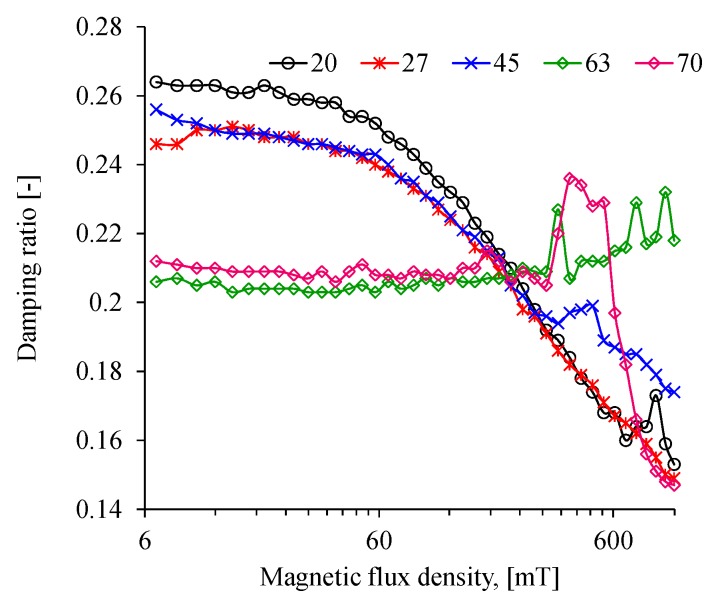
Influence of the magnetic flux density on the MRE damping ratio. There is a difference in the behavior of the MRE samples: for those with low CIP content (20, 27, and 45 wt %), the damping ratio values tend to decrease while for those with high CIP content (63 and 67 wt %), the damping ratio have almost a constant value up to a magnetic flux density of 311 mT.

Figure 18 shows damping ratio curves recorded during rheology tests. Note that the energy dissipation capacity of the composite MREs with the highest wt % of CIPs remains almost constant at increasing values of the magnetic flux, which starts to vary when the flux density exceeds the value of 311 mT. It can also be observed that the MRE with 63 wt % of CIPs slightly increases its damping ratio until we reach the magnetic flux intensity value of 1 T, however, the one with 70 wt % increases its damping ratio value up to a magnetic flux density of 311 mT and then, its value declines sharply as illustrated in Figure 17. The behavior exhibited by these MREs materials could be due to the scarcity of CIPs alignment, as observed in the microscopic analysis shown in Figure 7. Furthermore, the dissipation energy capacity for the MREs material samples reinforced with 20, 27 and 45 wt % of CIPs start to decline as soon as the magnitude of the induced magnetic flux density increases. Therefore, it is expected that these material samples will exhibit higher elastic behavior at increasing magnetic flux densities.

### 4.7. Loading and Unloading Uniaxial Magnetostatic Extension Tests

**Figure 19:**
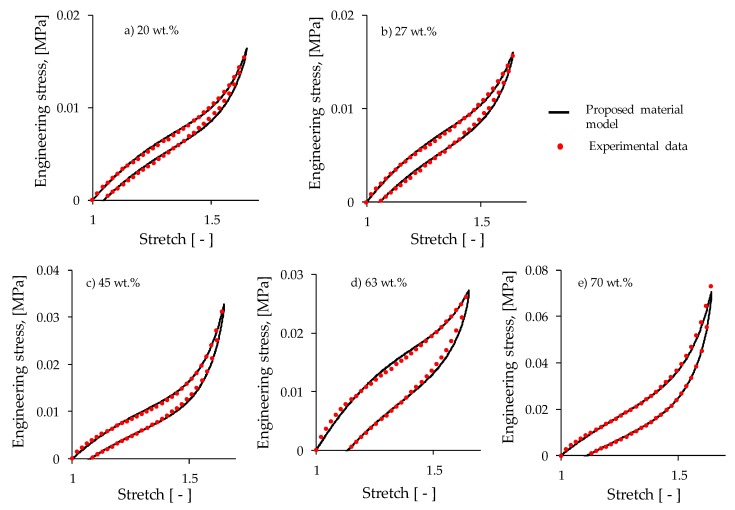
Collected experimental data of magnetorheological material samples subjected to loading and unloading cycles under the action of a magnetic flux density value of 52.2 mT. Notice that for increasing wt % of CIPs, the material shear moduli, the engineering stress, the dissipation of energy, and residual strains tend to increase.

**Figure 20:**
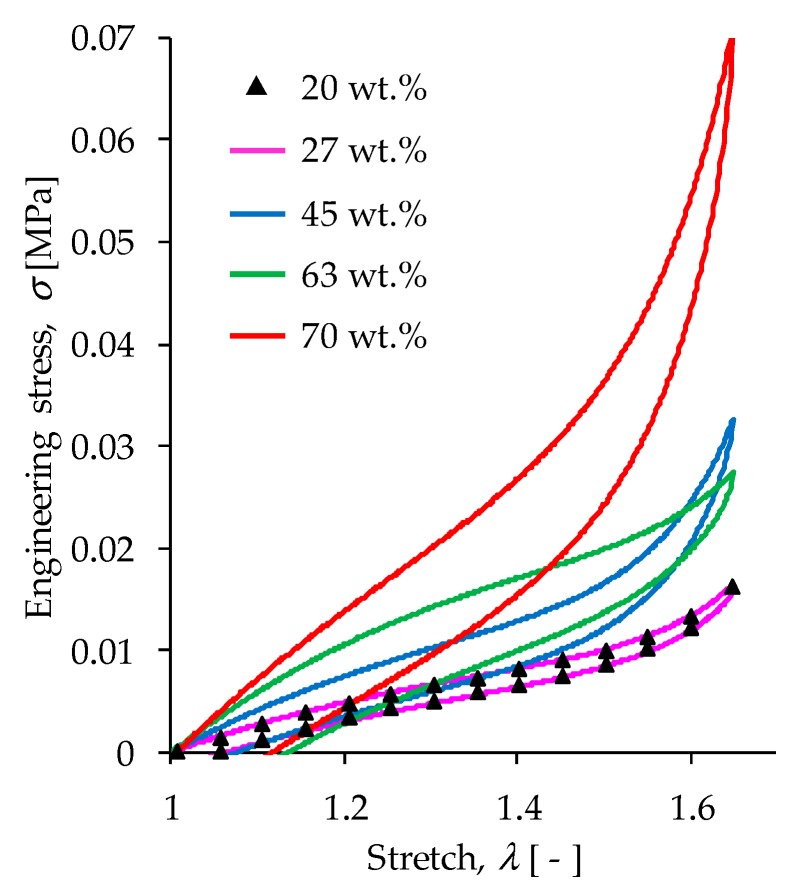
Theoretical predictions computed by using Equations (10) and (12) for different wt % of carbonyl iron particles added to the PDMS elastomeric matrix material under the action of a magnetic flux density of 52.2 mT. For the material samples with 20 and 27 wt % of CIPs, the mechanical response due to the magnetic flux intensity and applied load is almost the same because the volumetric fraction of CIPs used to develop the MRE material is almost the same, as illustrated in Table 1. As expected, the MRE samples developed by using 70 wt % of CIPs exhibit the highest shear modulus, dissipation energy, and engineering stress values. These results agree with rheological tests discussed in Section 4.6.

Loading and reloading tests were performed by using the tensile test set up shown in Figure 4. The dumbbell-shaped specimens were first subjected, without magnetic flux, to two cycles of pre-conditioning up to a pre-selected extension stretch of λ = 1.64 in order to have the sample subjected to uniform magnetic flux density, and to eliminate softening and permanent set effects. Afterwards, each material sample was subjected to loading and unloading cycles under the influence of magnetic flux density values of 11.1, 26.3, 39.5, and 52.2 mT, respectively, in order to evaluate the material responses due to magnetic phenomena. The collected experimental data and theoretical predictions obtained from the derived constitutive Equations (10) and (12) are illustrated in Figure 19 for the magnetic flux density of 52.2 mT. Notice in Figure 19, that the magnetic phenomena induced on the material samples′ softening and permanent set, modifies the samples′ mechanical properties depending on the wt % of CIPs. In fact, it can be observed in Figure 19 and Figure 20 that for increasing wt % of CIPs, the engineering stress, shear modulus, and residual strains tend to increase when the samples are subjected to a maximum stretch value of λ =1.64. At that stretch value, the material sample reinforced with 70 wt % of CIPs exhibits an engineering stress value that is about two times higher than those values exhibited by the other material samples, also, it experiences the highest stress-softened behavior.

The material parameters used in the proposed constitutive equations, *G*, *N*, *A*₁, *A*₂, *b*, *c* and *f* are listed in Table 1. Here, the solid black lines describe theoretical predictions, while the colored dots represent experimental data. Based on the computed theoretical predictions shown in Figure 19 and Figure 20, it was concluded that the developed constitutive equations for modeling magnetorheological materials subjected to both, elastic and magnetic phenomena, capture well both the qualitative and quantitative material response behavior in spite of having neglected the viscoelastic effects.

**Figure 21:**
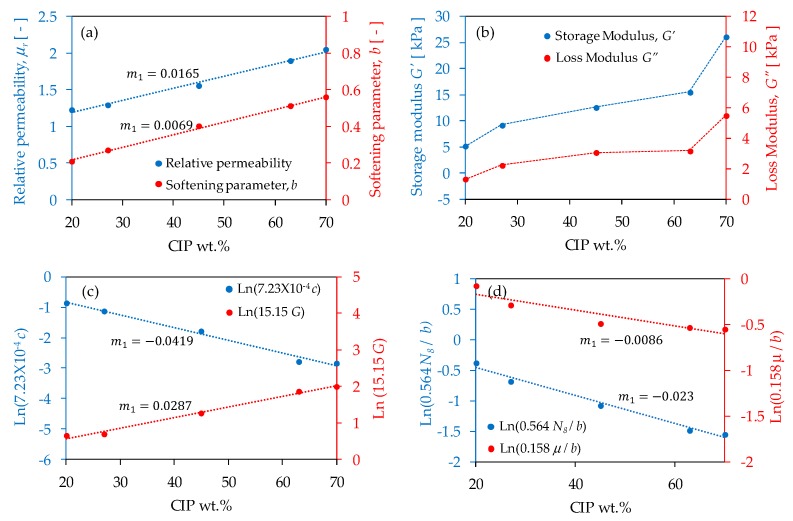
Magneto-mechanical charts for a constant magnetic flux density of 52.2 mT. (**a**) Linear relationship of the relative permeability and of the stress softening parameter versus the wt % of CIPs concentration. (**b**) Storage and loss moduli curves obtained from rheological measurements versus the iron particles concentration. (**c**) Semi-log curves of the residual strain parameter and the material shear modulus versus wt % of CIPs. Notice that both curves exhibit a constant slope that is an indication of the exponential material behavior when subjected to a magnetic field. (**d**) This figure illustrates the ratio of the $N_{8}/b,$ and μ/b plotted versus the wt % of CIPs. These curves indicate their exponential relationship with respect to the carbonyl iron particles concentration.

Figure 21 plots the variations of the mechanical and magnetic parameters versus the wt % of CIPs to identify possible relationships among them that may be used for design purposes. Notice in Figure 21a that the magnetic relative permeability linearly varies with respect to the wt % of CIPs concentration. Similar behavior was observed by Gorodkin et al. [39] when the measured specific susceptibility was plotted versus the wt % of carbonyl iron powders dispersed in MR fluids. Also, Figure 21a exhibits a linear relationship when plotting stress softening parameter values versus the wt % of CIPs. This implies that at increasing wt % of CIPs, the material can dissipate more energy when subjected to the combined effects of cyclic loading and magnetic flux density. This agrees with the stress-stretch curves shown in Figure 20 and the evolution of the loss modulus illustrated in Figure 21b. Figure 21c shows the semi-log curves of the residual strains constant, *c*, and the shear modulus versus the wt % of CIPs. Once again, there is a linear variation. It is also observed in Figure 21c, the influence that the CIPs has on the material mechanical response behavior because by increasing the concentration of iron particles added into the polymeric matrix, the composite material stiffened and permanent set effects diminish. Finally, the linear relationship between the permeability, chain number of links, and the stress softening parameters is confirmed in Figure 21d in which the semi-log plots of the ratios of $N_{8}/b,$ and μ/b versus the wt % of CIPs were calculated. These results can assist in the design and fabrication of components made from MREs with different concentration of CIPs, when subjected to external loads and magnetic effects.

## 5. Conclusions

The design and fabrication of a solenoid device to generate uniform magnetic flux density was a key factor to successfully produce MRE samples for different concentrations of CIPs. Finite element computer simulations were performed to identify the position of the specimen within the coil at which the device provides uniform magnetic flux density. Then, we used this device to fabricate the MRE samples with CIPs aligned along the longitudinal axis of the manufactured parts. Because the magnitude of the induced magnetic forces was limited to a magnetic flux intensity of 52.2 mT, the samples with 63 and 70 wt % of CIPs did not achieve particle alignment. Experimental characterization of the fabricated MRE samples were performed in order to assess particles′ morphology, distribution, and alignment within the PDMS matrix. We also used SEM images to confirm that CIPs were wrapped in PDMS matrix without phase separation because of the percentage of SO used to manufacture the material samples.

On the other hand, rheological test measurements showed a change in the relative MR effects for the MRE reinforced with 70 wt % of CIPs when a magnetic flux density of 1093 mT was applied. It was also found that the damping ratio values tend to remain constant for concentrations of 63 and 70 wt % of CIPs when the applied magnetics flux densities do not exceed the values of 311 mT. For these materials, the energy dissipation potential remains almost constant due to the isotropic distribution of the CPIs in the elastomeric matrix material. However, for concentrations of 20, 27 and 45 wt % of CIPs, the damping ratio of the anisotropic MREs decreases for increasing values of the magnetic flux density.

During uniaxial experimental tests, the samples were stretched to a maximum value of 1.64 to subject the samples to a uniform magnetic flux density within the coil. The collected data showed that for the samples made with 63 wt % of CIPs, the MRE stiffness attains the highest increase in its magnitude value when subjected to different magnetic flux density values. To quantify the impact that the magnetic flux density has on the material stiffness when this is subjected to tensile tests, the stiffness magnetorheological effect concept was defined. This SMR effect allowed us to identify that the MRE with 63 wt % of CIPs has a SMR effect of 118% when a 52 mT magnetic flux density is applied, as shown in Figure 14. An increase in the relative storage and loss MR effects of 2282% and 1548%, respectively, was observed in the MRE samples when subjected to a magnetic flux density of 1093 mT.

To study Mullin′s effects due to elastic and magnetic phenomena, the MRE samples were first subjected to a two cycles of pre-conditioning tests without magnetic flux in order to reduce softening effects due to the manufacturing process of the material samples. Then, each material sample was subjected to loading-unloading cycles under the action of an increasing longitudinal magnetic flux intensity per loading-unloading cycle. The collected data were used to assess the accuracy of the constitutive material model that was developed to predict softening and permanent set of MRE under the combined action of elastic and magnetic effects. A comparison of theoretical predictions and experimental data showed that our proposed magneto-elastic constitutive material model, with only six material constants that need to be fitted, predicts well the qualitative and quantitative behavior of the MRE samples in spite of neglecting viscoelastic effects, besides, constitutive Equations (10) and (12) can be adapted to model hard-magnetic soft materials just by adding slight changes. These model adjustments will be addressed in a forthcoming paper to be submitted elsewhere.

Based on theoretical predictions, a linear relationship was found between magnetic permeability, stress softening, permanent set, and material stiffness versus the concentration of carbonyl iron particles. These findings can be used to develop MREs with a certain wt % of CIPs to promote the desired magneto-mechanical properties. Therefore, we believe that the design charts shown in Figure 21 could assist designers in identifying how to produce a magnetorheological elastomer with the desired magneto-mechanical response behavior.

Finally, and to the best of the authors′ knowledge, this is the first material model that addresses stress-softening and permanent set effects of MRE subjected to both elastic and magnetic phenomena which provides good theoretical predictions when compared to experimental data.

## Figures and Tables

**Table 1:** Material constants used to fit the cyclic experimental data. Applied magnetic flux density of B= 52.2mT, with a magnetic permeability of $\mu =\mu_{r}\;\mu_{0}$, and vacuum magnetic permeability equals to $\mu_{0}=4\pi 10^{\left(-7\right)}\;H/m.$ Notice that there is a linear relationship between the wt % of CIPs versus the magnetic permeability values.

CIPs wt %	*G* (MPa)	*N*_8_ (-)	*A*_1_ (MPa)	*A*_2_ (MPa)	*b* (-)	*c* (MPa)	*f* (-)	*μ_r_* (-)
20	0.0126	1.550	881.305	−0.48	0.21	580	0.034	1.23
27	0.0132	1.560	840.350	−0.33	0.27	450	0.049	1.29
45	0.0237	1.495	699.355	−0.27	0.40	230	0.103	1.55
63	0.0424	1.700	573.520	−0.20	0.51	85	0.192	1.89
70	0.0481	1.475	528.800	−0.11	0.58	80	0.247	2.05
